# Construction and application of human neonatal DTI atlases

**DOI:** 10.3389/fnana.2015.00138

**Published:** 2015-10-26

**Authors:** Rajiv Deshpande, Linda Chang, Kenichi Oishi

**Affiliations:** ^1^Department of Radiology, Johns Hopkins UniversityBaltimore, MD, USA; ^2^Biomedical Engineering, Johns Hopkins UniversityBaltimore, MD, USA; ^3^Department of Medicine, School of Medicine, University of Hawaii at ManoaHonolulu, HI, USA

**Keywords:** atlas-based analysis, diffusion tensor imaging, neonatal brain atlas, probabilistic, parcellation map, tractography

## Abstract

Atlas-based MRI analysis is one of many analytical methods and is used to investigate typical as well as abnormal neurodevelopment. It has been widely applied to the adult and pediatric populations. Successful applications of atlas-based analysis (ABA) in those cohorts have motivated the creation of a neonatal atlas and parcellation map (PM). The purpose of this review is to discuss the various neonatal diffusion tensor imaging (DTI) atlases that are available for use in ABA, examine how such atlases are constructed, review their applications, and discuss future directions in DTI. Neonatal DTI atlases are created from a template, which can be study-specific or standardized, and merged with the corresponding PM. Study-specific templates can retain higher image registration accuracy, but are usually not applicable across different studies. However, standardized templates can be used to make comparisons among various studies, but may not accurately reflect the anatomies of the study population. Methods such as volume-based template estimation are being developed to overcome these limitations. The applications for ABA, including atlas-based image quantification and atlas-based connectivity analysis, vary from quantifying neurodevelopmental progress to analyzing population differences in groups of neonates. ABA can also be applied to detect pathology related to prematurity at birth or exposure to toxic substances. Future directions for this method include research designed to increase the accuracy of the image parcellation. Methods such as multi-atlas label fusion and multi-modal analysis applied to neonatal DTI currently comprise an active field of research. Moreover, ABA can be used in high-throughput analysis to efficiently process medical images and to assess longitudinal brain changes. The overarching goal of neonatal ABA is application to the clinical setting, to assist with diagnoses, monitor disease progression and, ultimately, outcome prediction.

## Introduction

Diffusion tensor imaging (DTI) is a magnetic resonance imaging (MRI) modality that exploits the water diffusion (Brownian motion of water molecules) to compute a tensor, from which various parameters can be calculated. DTI has been developed and used in human brain research since the 1990s, and it still offers many useful features and advantages. DTI is capable of delineating three-dimensional white matter anatomy with high fidelity, and the parameters derived from DTI, such as fractional anisotropy (FA), mean diffusivity (MD), axial diffusivity (AD), and radial diffusivity (RD), and can potentially indicate the axonal organization, degree of myelination, fiber coherence, and axonal density, which, altogether, can reflect the developmental status of the brain (Beaulieu, 2002; Huppi and Dubois, 2006). Among various MRI modalities based on water diffusion, DTI can be acquired in short scan times with relatively higher signal-to-noise ratio, which is advantageous in clinical studies or studies of babies and the pediatric population, because such short DTI scans minimizes the potential motion that often occur during longer scans, which is particularly prevalent in individuals with illnesses or in infants and children.

Quantification of DTI and other modalities in general, is an important process that permits the introduction of statistical analysis. Various methods are used for the quantification of DTI, and the simplest approach is to place regions-of-interest (ROIs) on DTI to measure the parameters of selected anatomical structures. This approach is accordingly useful for hypothesis-testing research, because the ROIs are selected based on an *a priori* hypothesis, and the effect of a disease or condition on the selected ROIs can be investigated. However, this ROI-based approach is not suitable for investigations of anatomical areas where the effects of a disease or condition are initially unknown. For such studies, whole-brain analysis is the method of choice.

**FIGURE 1:**
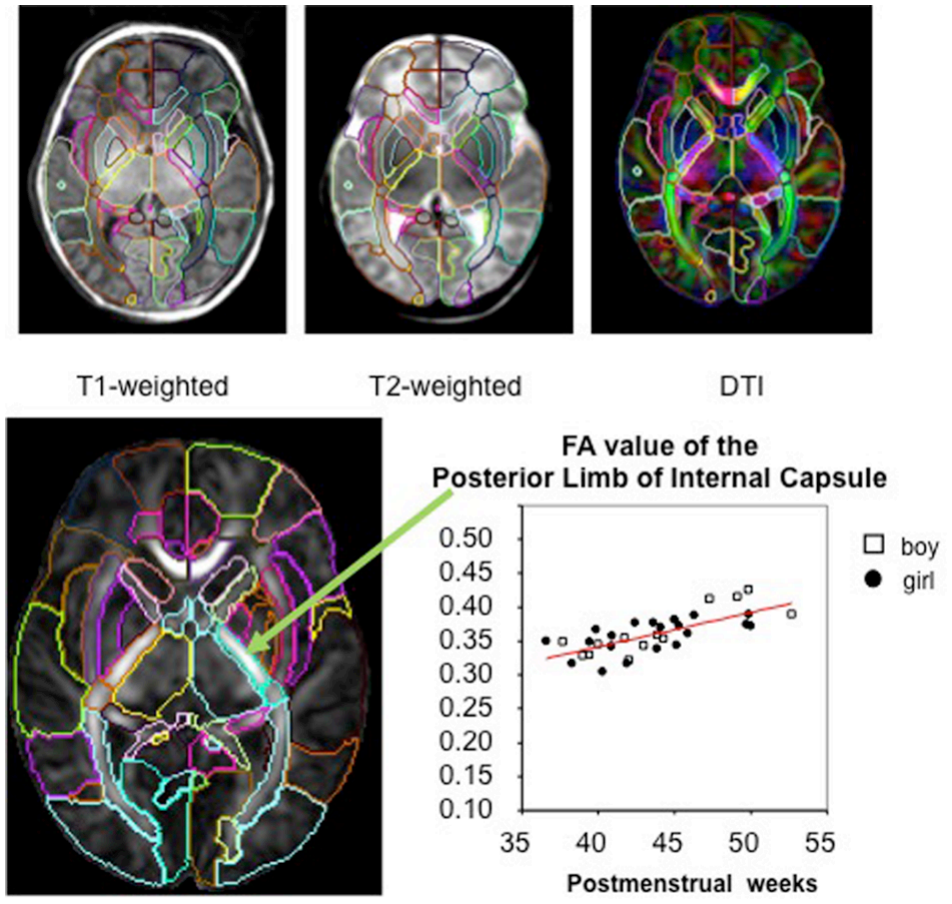
**The parcellation map (PM) segments the brain into various structures and permits parameter-based analysis, such as measurement of the FA value, at the structural level on images from various modalities (used with permission from Oishi et al., 2013)**.

**FIGURE 2:**
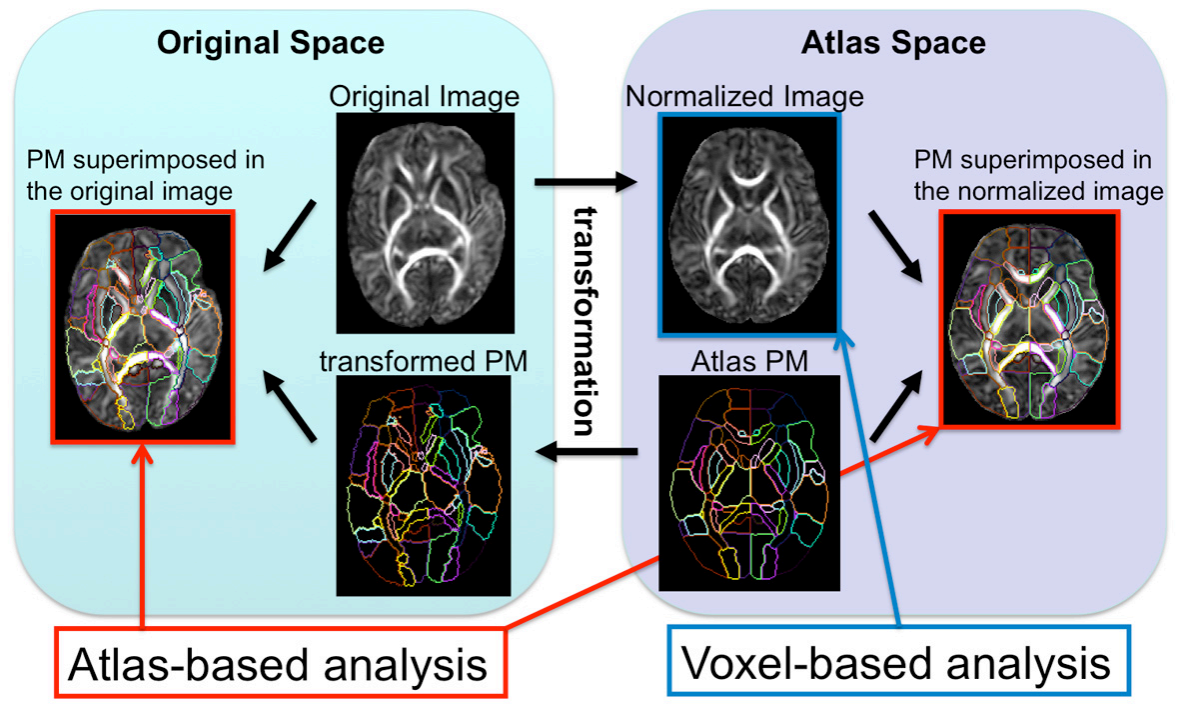
**The image transformation pipeline normalizes original subject images to the atlas space and reverse transforms the atlas’s PM to apply back onto the subject image**.

In whole-brain analysis, a template is often used as a target image and subject images are mathematically transformed in order to register the brain structures of each individual to those of the template space. The transformation of the image to the template is called “normalization.” Since each brain is different in terms of size, shape, and proportion, a template is necessary to normalize these features to perform a voxel-by-voxel analysis of the DTI parameters. After image normalization, comparison between groups of subjects (e.g., normal vs. diseased groups) or regression to non-image parameters (e.g., age, clinical status, or function) can be performed on a voxel-by-voxel basis, which is known as voxel-based analysis (VBA). In addition to the VBA, the “parcellation map,” (PM), a set of pre-defined anatomical boundaries of the template brain, is used for a parcel-by-parcel statistical analysis (**Figure 1**). Here, each parcel groups the voxels together to increase the signal-to-noise ratio by averaging signals inside the parcel, and to streamline the neuroscientific or clinical interpretation of the statistical results. Parcel-by-parcel statistical analysis is also applicable to analysis in the individual space. This can be achieved simply by transforming a PM from a template space onto individual images (**Figure 2**). Currently, there are more sophisticated variations, including a multi-atlas label fusion method (Tang et al., 2014).

This parcel-by-parcel analysis, either in a template space or individual space, is called “atlas-based analysis (ABA),” especially when a standard brain atlas, such as the MNI or ICBM atlas^1^^,^^2^, with the corresponding PM, is used as the template. The use of a standard atlas is advantageous to facilitate communication and comparisons of brain imaging data among different institutions. There are various types of atlases and PMs, as listed on the websites of the Oxford Center for Functional Magnetic Resonance Imaging of the Brain^3^, the Laboratory of Neuro Imaging^4^, the University of North Carolina Image Display, Enhancement, and Analysis Group^5^, the Athinoula A. Martinos Center for Biomedical Imaging, Laboratory for Computational Neuroimaging^6^, the Research Center Jülich, Institute of Neuroscience and Medicine^7^, or the Johns Hopkins Laboratory of Brain Anatomical MRI^8^^,^^9^, which are used for different research applications. Various PMs accentuate the unique features of the brain, such as structural units, vascular territory, anatomical connectivity, functional connectivity, and cytoarchitecture. These diverse PMs allow researchers to examine the different ways neurological diseases can affect the brain.

**FIGURE 3:**
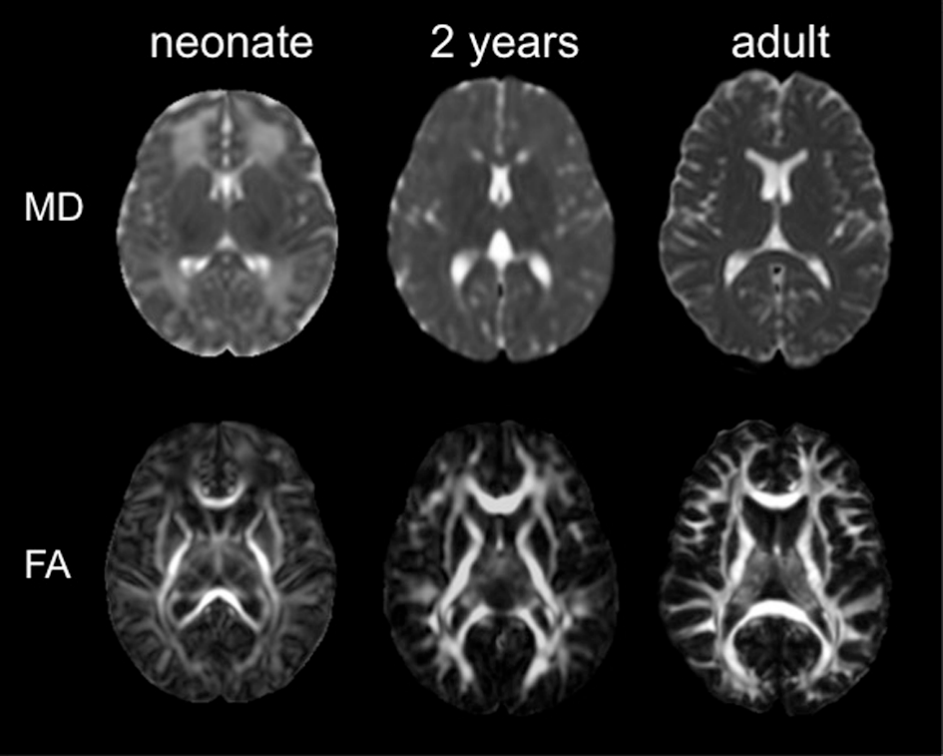
**The mean diffusivity (MD) map and the fractional anisotropy (FA) map of a neonate, a 2 years child, and an adult (used with permission from Oishi et al., 2013)**.

The ABA has been applied to DTI of pediatric and adult populations to investigate normal neurodevelopment as well as the abnormalities caused by various diseases, such as cerebral palsy (Faria et al., 2010; Yoshida et al., 2013), Williams syndrome (Faria et al., 2012b), and Rett syndrome (Oishi et al., 2013). The successes of these researches have engendered the application of ABA to study neurodevelopment in earlier ages, especially in perinatal–neonatal brains. However, application of adult or pediatric brain atlases to neonatal brain research is not straightforward, because there are substantial differences in cytoarchitecture and myelination between neonatal brains and older brains that can cause differences in DTI-derived contrasts (**Figure 3**). Therefore, DTI atlases with corresponding PMs specifically created for the neonatal brain are often used for ABA of the perinatal–neonatal population. Some of the various types of publicly available atlases and PMs that have been applied to neonatal DTI studies are summarized in **Table 1**.

**Table 1:** Atlases applied to neonatal DTI studies.

Name of atlas	Description/parcellation map (PM) availability	Contrasts	Age range	Reference
University of North Carolina-Chapel Hill Brain Atlas	Atlas components include intensity models, tissue probability maps. Uses an anatomical PM	T1w and T2w	Neonates, 1 years, and 2 years	Brown et al., 2014
4D Imperial College London Neonatal Brain Atlas	Dynamic, probabilistic atlas for stages (29–44 weeks gestational age) in neonatal brain development	T1w and T2w	Neonate	Brown et al., 2014
JHU-MNI Single-Subject Brain Atlas (Eve Atlas)	Provides co-registered T1, T2, and DTI images from a single subject. Uses a white and gray matter anatomical PM	T1w, T2w, and DTI	Adult	Djamanakova et al., 2014 Faria et al., 2010 Geng et al., 2012 Melbourne et al., 2014 Tang et al., 2014
ICBM DTI-81 Brain Atlas	Stereotaxic and probabilistic white matter atlas integrating DTI-based data with ICBM 152 template. Uses a white matter anatomical PM	DTI	Adult	Fonov et al., 2011 Klein et al., 2010 Miller et al., 2013 Oishi et al., 2011a Oishi et al., 2009
JHU Neonatal Brain Atlas	Group averaged and single subject brain atlas of the neonatal brain that integrates DTI-data with co-registered anatomical MRI. Uses a white and gray matter anatomical PM	T1w, T2w, and DTI	Neonate	Oishi et al., 2011b Pannek et al., 2013 Ratnarajah et al., 2013 Rose et al., 2014a Rose et al., 2014b Kersbergen et al., 2014 Zhang et al., 2014


In this review, currently available neonatal DTI atlases are introduced, along with studies that applied these atlases.

## Neonatal DTI Atlases

The initial step for the ABA comprises the creation of a template (or a set of templates) and the corresponding PM. There are two types of template images: one in a study-specific space and one in the standardized coordinates. Study-specific templates retain the average features of the study population and are advantageous for accurate image normalization (Hamm et al., 2009; Tang et al., 2009; Klein et al., 2010; Fonov et al., 2011; Jia et al., 2011). However, standardized templates are particularly valuable when the specific brain regions will be compared across different studies.

Two studies are examples of pioneering work in study-specific DTI templates (Marc et al., 2010; Wang et al., 2012); both used an unbiased diffeomorphic atlas-building method based on a non-linear high-dimensional fluid deformation. Namely, they first created an initial FA template, which was intensity-histogram-normalized. Then, non-linear transformations were applied to the initial template to produce a deformation field for each image. All the tensor images were then reoriented into the unbiased space using the finite strain approximation. The atlas was then developed by averaging all the reoriented tensor images in log-Euclidean space. Once template is created and DTIs are normalized to the template space, Tract-Based Spatial Statistics (TBSS) has been widely used for the VBM, to enhance sensitivity by focusing on the improved registration accuracy of the core white matter voxels. One TBSS approach was optimized for neonates by reducing registration errors through the identification of an appropriate single-subject FA map from the study population, followed by the creation of a study-specific FA map template (Ball et al., 2010).

After creating the template, the next step is to construct the corresponding PM. Producing a PM can be accomplished manually (Wang et al., 2012), but unless the PM covers the whole brain, the manually drawn PM is suitable only for the hypothesis-driven studies. One can also warp a whole-brain gray matter PM from a standard non-DTI template to the study-specific template, such as that employed using the University of North Carolina Chapel Hill neonatal atlas (Brown et al., 2014). This approach was effective for the analysis of anatomical connectivity among cortical structures; however, since the white matter structures were not fully parcellated, structure-by-structure analysis of the white matter was outside the focus of that study. For such studies, whole-brain PM, including white matter structures, is required. Nevertheless, generating a whole-brain PM for a study-specific DTI template is laborious and time-consuming, as it needs to cover the entire brain three-dimensionally, and manual drawing requires professional anatomical knowledge.

**FIGURE 4:**
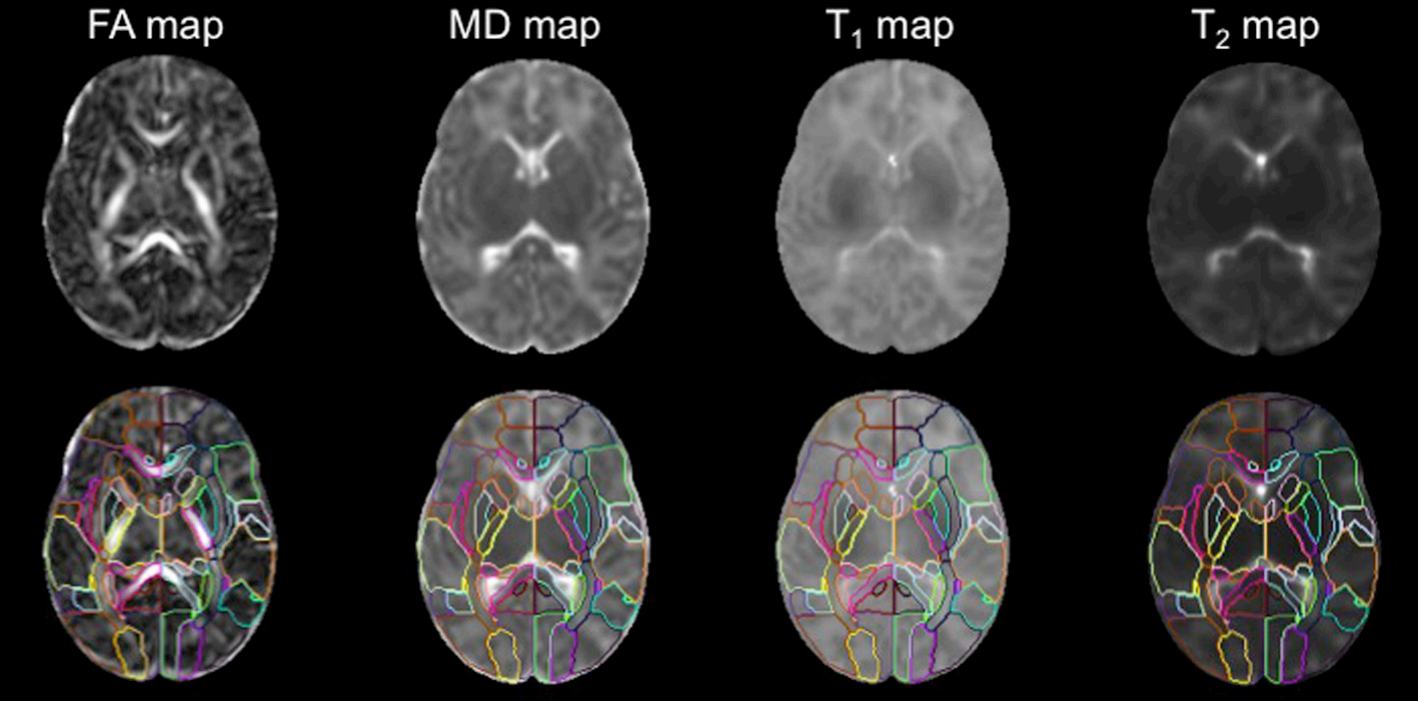
**The DTI, T1, and T2 maps were transformed from the subject space to the atlas space via co-registration and normalization.** The application of the PM permits analysis of FA,MD, T1, and T2 values at the structural level (used with permission from Oishi et al., 2011b).

Using a standard template and the corresponding PM is beneficial for DTI studies, because the white matter area, which looks homogeneous on conventional T1- and T2- weighted images, can be parcellated into numerous fiber bundles (**Figure 4**). In addition, since defining anatomical boundaries is often ambiguous, using a standard PM can increase reproducibility in defining anatomical regions. Templates from the Montreal Neurological Institute (MNI) and the International Consortium of Brain Mapping^10^ (ICBM) have long been used as the standard template for adult brain images. PMs from these templates are well-developed and can be applied as a tool for ABA.

Creating the ICBM atlas and the single-subject atlas follows a protocol that is highlighted by the alignment of the anterior commissure–posterior commissure (AC-PC) line. Using similar steps, a set of neonatal DTI atlases was constructed with co-registered T1- and T2-weighted atlases (Oishi et al., 2011b). The set included the Johns Hopkins University neonate linear (JHU-neonate-linear), non-linear (JHU-neonate-non-linear), and single-subject (JHU-neonate-SS) atlases^11^. The JHU-neonate-linear and the JHU-neonate-non-linear atlases are group-averaged images based on co-registered T1- and T2- weighted DTI images acquired from normal term-born neonates. The JHU-neonate-linear was created using only linear transformations, such as rotation, translation, scaling, and shear, among the component images. The large deformation diffeomorphic metric mapping (LDDMM; Ceritoglu et al., 2009) was used to create the JHU-neonate-non-linear atlas. The JHU-neonate-SS atlas was created from a representative single–individual image with the shape and size adjusted to represent population-averaged features. The associated PMs include both gray and white matter structures that cover the entire neonatal brain.

The possible drawback of using standard templates is that they may not necessarily represent the average anatomical features of the study population, although, for DTI without visible abnormalities, the study-specific template and the standardized template resulted in similar normalization accuracy, as demonstrated by (Zhang and Arfanakis, 2013). However, when the template is applied to a population with specific diseases, which typically demonstrates local alterations in volume and shape, the accuracy in image normalization is usually lower than when a study-specific template is applied. An attempt to overcome this limitation is the Volume-based Template Estimation (VTE) method (Zhang et al., 2014). VTE enables iterative modifications of the standard atlas toward the average of the study population to create a template and a PM that are customized for the study. This method can increase the accuracy of image normalization, especially in the cortical area of neonatal brains, while the boundary definitions of the PM are maintained at those of the JHU-neonate-SS atlas.

## Application of Neonatal Atlases

The neonatal atlas has been applied to perform ABA on neonatal brains. It has been used to measure parameters derived from DTI and to investigate connectivity among brain regions. Such applications demonstrate the potential utility of quantitative tools in neurodevelopmental assessments.

## Analysis of DTI-derived Parameters

### Brain Development

To examine developmental changes of the neonatal brain with age, the JHU-neonate-SS atlas was applied to full-term healthy neonates at 37–53 post-conception weeks (Oishi et al., 2011b, 2013). A multimodality approach that consists of DTI, T1-, and T2-maps was adopted to assess the microstructural development of 122 anatomical structures determined by the PM. Increases in FA and decreases in diffusivity, T1, and T2 with age were generally observed, congruent with the histopathological evidence of ongoing myelination and axonal development during this age-range that occurs in the posterior-to-anterior and central-to-peripheral directions (Kinney et al., 1988). Following this healthy neonatal DTI study, Rose et al. (2014a) examined whether these specific directions of brain maturation could be seen in very-low-birth-weight neonates as well. Sixty-six such neonates with no evidence of congenital brain abnormalities successfully underwent DTI scans and the JHU-neonate-SS atlas was used for the quantification. The posterior-to-anterior and central-to-peripheral pattern was again observed, especially in the corona radiata, corpus callosum, and internal capsule. Within projection and association fibers, centrally located fibers, such as the cerebellar peduncles and the posterior limb of the internal capsule, were also observed with greater FA values than those in peripheral areas, such as the superior longitudinal fasciculus or the external capsule. These studies demonstrated the possibility of creating a standardized growth percentile chart of brain development based on MRI- and DTI-derived parameters via ABA. However, since the study design was cross-sectional, information about intra-individual changes in MRI- or DTI-derived parameters was not provided.

To overcome the limitation of cross-sectional analysis, longitudinal design was adopted to investigate developmental changes during 30 to 40 post-conceptional weeks (Kersbergen et al., 2014). The JHU-neonate-SS atlas was used for the ABA to quantify FA and diffusivity measures from 40 preterm neonates without brain injury and with normal developmental outcome at 15 months of age. FA was found to have increased in 84 brain regions, most significantly in the posterior limb of the internal capsule, the cerebral peduncles, the sagittal stratum, the corona radiata, and other central structures. Conversely, FA values decreased most in the temporal and occipital cortical regions, while MD, AD, and RD values decreased in most brain regions.

### Genetic Effects

To assess how influential genetic and environmental factors could be on WM microstructure in neonates, Geng et al. (2012) analyzed the heritability of WM microstructures evaluated by DTI in healthy full-term neonate twins. The JHU-DTI-MNI (a.k.a. “Eve”) atlas (Oishi et al., 2009) with the PM was adjusted to the study-specific template to perform ABA of 98 anatomical structures in 173 neonates; 63 twin sets, and 47 unpaired twins were included. To assess the genetic and environmental effects on the DTI-derived parameters of each structure, a univariate twin modeling approach was applied (Neale and Cardon, 1992). Namely, effects of additive genetic factors and environment, shared or unshared by twin pairs, were modeled and fitted to test the significance of each effect on DTI measures. The results indicated that structures with high FA and low RD, which might be associated with high maturation at the time of the DTI scan, tended to show less influence of genetic effects on DTI than that of other structures. For example, the genetic contribution to the FA of right posterior limb of the internal capsule was 0.00, while the contribution of common environmental factors was 0.50. The study suggests that the strength of the genetic effect depends on the regional maturation status of the neonatal brain.

### Population Differences

To investigate differences in neuroanatomical features among ethnic groups, Bai et al. (2012), quantified brain size, morphology, and DTI parameters of Malay, Chinese, and Indian neonates. The JHU-neonate-SS was iteratively transformed to construct a study-customized atlas to evaluate 177 neonates. Indian neonates had more elongated brains despite similar brain volume among the three ethnicities. Compared to Indian neonates, the Malay neonates had lower FAs in the left anterior limb of the internal capsule, the left thalamus, the anterior corpus callosum, and the left midbrain, while the Chinese neonates had only lower FA in the anterior part of the corpus callosum. These results suggest that ethnicities should be considered when performing a statistical comparison of the DTI-derived parameters among groups.

### Effects of Prematurity and Related Risk Factors

Prematurity is one amongst a host of perinatal stressors that is known to adversely affect neurological development in neonates. Preterm birth is also related to the occurrence of other risk factors that might affect brain development. Therefore, it is important to investigate the respective contributions of prematurity and other risk factors to explain developmental alterations that occur in babies born preterm. The effects of other medical factors, including bronchopulmonary dysplasia, retinopathy of prematurity, necrotizing enterocolitis, sepsis, and serum levels of C-reactive protein, albumin, glucose, and bilirubin, were studied (Rose et al., 2014b). The JHU-neonate-SS atlas was used for the ABA on a cohort of 66 very-low-birth-weight preterm infants who underwent DTI scans at a term-equivalent age. Among anatomical structures, the posterior thalamus MD was associated with lower gestational age at birth and lower levels of albumin and bilirubin. These findings imply that the thalamus may be a structure vulnerable to prematurity and accompanying risk factors. However, interactions among brain abnormality, prematurity, and risk factors still need to be demonstrated.

### Effect of *In Utero* Exposure to Stimulants

**FIGURE 5:**
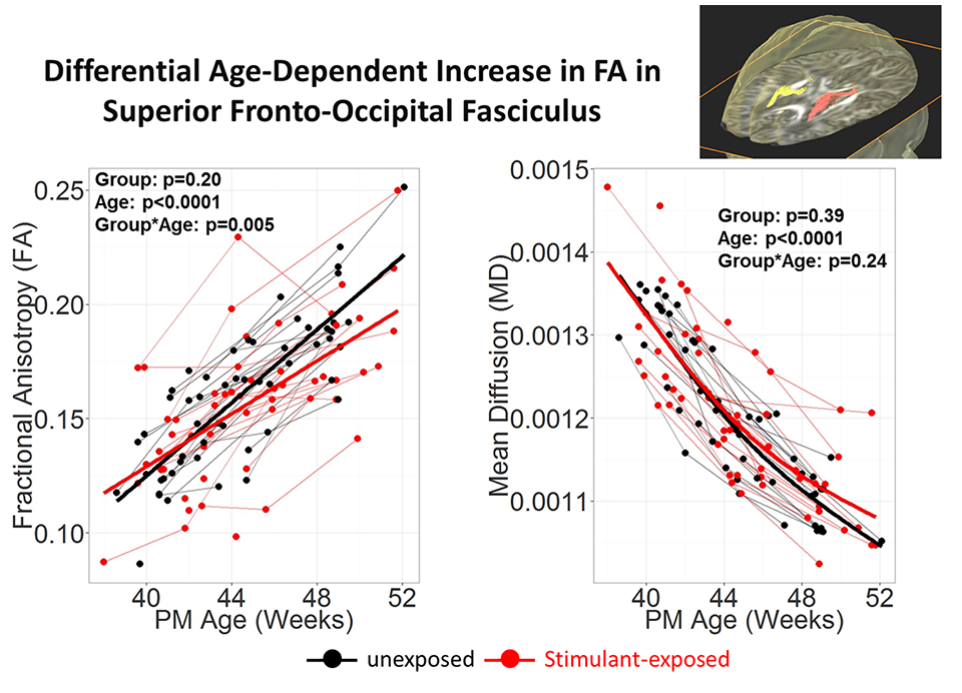
**Stimulant (Methamphetamine and tobacco)-exposed infants (*n* = 25) had less steep age-dependent increase in FA than the unexposed infants (*n* = 25) in the superior fronto-occipital fasciculus.** Each infant was scanned 2–3 times during the first 3 months of life. Repeated measures analysis using mixed model with resolution, sex and Index of Social Position as covariates. PM Age = postmenstrual age (from poster presesentation at Neurobehavioral Teratology Society by Chang et al., 2012).

To investigate the effects of prenatal exposure to stimulants, such as methamphetamine or nicotine, DTI scans were acquired longitudinally from babies who were prenatally exposed to stimulants, and the results were compared to those from babies without any prenatal stimulant exposure (Chang et al., 2012). The JHU-neonate-SS atlas was applied for the ABA to quantify the FA, MD, AD, and RD of each structure. Although conventional T1- and T2- weighted images of these babies were normal appearing, the age-related increase in FA tended to be lower in stimulant-exposed infants, especially in the superior fronto-occipital fasciculus (**Figure 5**), which suggests a slower rate or delayed myelination in stimulant-exposed babies. This indicated the potential for ABA to detect anatomical alterations related to stimulant exposure, although relationships between detected anatomical alterations and later neurological, behavioral, and cognitive functional outcomes need to be elucidated.

## Analysis of Brain Connectivity

Diffusion tensor imaging, analyzed with graph theory, is useful in non-invasively mapping and assessing neural networks (connectome) of the brain in adults (for review, Lo et al., 2011; Griffa et al., 2013). Brain atlases and the PMs are often used to parcellate the brain into local areas that serve as the network nodes (de Reus and van den Heuvel, 2013; O’Donnell et al., 2013). Although the application to investigate developmental changes is still challenging (for review, Tymofiyeva et al., 2014), an increasing number of studies are investigating connectomes of neonatal brains, some of which are introduced below.

### Asymmetrical Neural Network Development

The human brain is structurally asymmetrical, a feature that reflects its diverse, specialized functions. However, it has not been well-established whether such asymmetries can be traced back to the perinatal period. This was one of the aims in a study (Ratnarajah et al., 2013) that acquired DTI in 124 healthy, full-term neonates, and the FA maps were transformed to the JHU-neonate-SS atlas for the image parcellation. For whole-brain tractography, the fiber-assignment by continuous tracking (FACT) algorithm (Mori et al., 1999) was used to compute the fiber trajectories that connect between parcels. In order to characterize the brain, the fibers were analyzed for small-worldness, type of efficiency (global or local), and betweenness-centrality. Small-worldness defines a network wherein any two nodes are not necessarily neighbors, but are relatively well-connected (Watts and Strogatz, 1998). Global efficiency describes how efficiently information can pass through the network (Latora and Marchiori, 2001). Local efficiency indicates how well information can be exchanged in a local neighborhood, or subnetwork (Rubinov and Sporns, 2010). Betweenness-centrality is a statistic that identifies the most central nodes of a network (Freeman, 1978). These values were used altogether to calculate a lateralization index (LI), a proxy for brain asymmetry. The analysis on connectivity revealed that, in neonates, both cerebral hemispheres exhibited small-world characteristics, and that the LI was higher in the left hemisphere. This suggested that the left hemisphere of neonates was better equipped to balance local necessities and wide-range interactions. Furthermore, the study insinuates that the neonatal brain favors a local flow of information and seeks to reduce the number of long-distance connections. This small-world preference of the neonatal brain has also been observed in the brains of adults (Gong et al., 2009), which suggests that the brain provides an efficient network from birth. In addition, LI values indicate a significantly leftward tendency in the precentral gyrus, the precuneus fusiform, the entorhinal cortex, and the insular cortex, and, likewise, a rightward tendency in the gyrus rectus, the cingulate gyrus, the hippocampus, and the putamen. Such LI values indicated that those structures communicated more efficiently in their respective hemispheres, demonstrating a betweenness-centrality asymmetry in the neural network. These results suggest that the brain already exhibits asymmetry at birth.

### Effect of Prematurity at Birth

The effect of prematurity was also examined from a network-based perspective (Pannek et al., 2013). To investigate the effect of prematurity, cortico-cortical connections in 18 preterm and nine full-term babies were compared. The JHU-neonate non-linear atlas was used as an initial template to create a study-specific FA map upon which the structural PM could be applied, and to define 24 cortical regions as nodes for connectivity analysis. Network-based statistical analysis was then performed to compare DTI-derived measurements (FA and MD) and T2 values between the cohorts. In all, 433 connections were analyzed. FA was lower in preterm neonates than in full-term controls in a network of nodes whose central nodes were the superior temporal lobe, the left fusiform gyrus, and a network of connections between the frontal and motor nodes. MD was found to be higher for preterm infants in a majority of the connections, leading to a global elevation of diffusivity. T2 was higher in the preterm group in a network that included the left superior frontal lobe, the left cingulate gyrus, and the right precuneus. Although there were no correlations between gestational age at birth and DTI-derived measurements, a negative correlation between T2 and gestational age at birth was identified in a network that included the bilateral precuneus, the left lateral orbito-frontal gyrus, the left middle temporal gyrus, the left superior temporal gyrus, and left lingual gyrus. This study demonstrated the possible influence of prematurity on brain microstructure, as assessed by DTI and T2, as well as the utility of multimodal analyses to detect different developmental processes in the neonatal brain.

### Relationship between White Matter Connections and the Cortical Folding Patterns

The cortex has developed over evolutionary time an exquisitely complex folding pattern, which is unique to the human brain. The role and nature of cortical folding still remains controversial, although the folding of the brain was hypothesized as a natural consequence of connectivity to subcortical areas (Van Essen, 1997). To investigate correlations between white matter connections and the cortical folding pattern, one study quantified the amount of cortical connectivity through tractography and the local cortical folding patterns through ABA of the gyrification index, as well as the sulcation ratio using the JHU-neonate atlas (Melbourne et al., 2014). The white matter connectivity correlated specifically with the sulcation ratio of the overlying cortex, suggesting that the gyrification pattern represents the development of the underlying white matter. As demonstrated, the ABA is especially useful for the integration of information from multiple modalities.

## Future of Neonatal DTI Atlas

### Multi-atlas-based Structural Parcellation

Conventional ABA methods usually utilize a single atlas. However, the limitation of a single-atlas approach is that the template does not necessarily represent the features of a study population. The possibility of voxel mismatch due to differences in contrast and morphology between the subject image and the template image is a factor that can contribute to inaccuracy in the parcellation. In order to overcome this limitation, a multi-atlas label fusion method has been developed and applied to adult brain images. In this approach, multiple atlases with different anatomical features are transformed to the target image, and the anatomical labels based on the transformed PMs are fused to define a target-specific PM. Studies have been performed to assess the accuracy of parcellations via multiple atlas fusion protocols, and have reported higher accuracy than single-atlas methods in the adult and pediatric populations (Heckemann et al., 2006; Aljabar et al., 2009; Tang et al., 2013, 2014). Such successes encourage the application of such methods to the neonatal population and to create accurate PM for each brain.

### Multi-modal Analysis

A multi-modal approach rather than a single-modality approach has been shown to be superior in detecting abnormalities related to neurological diseases (Allder et al., 2003; Brockmann et al., 2003; Rostasy et al., 2003; Kauczor, 2005; Wiest et al., 2005; Oishi et al., 2011a). Typically, the different modalities complement each other, and, together, can permit more detailed interpretation of the brain. However, one of the biggest challenges for multi-modal analysis is to establish a common anatomical framework that can integrate intra-subject as well as cross-subject multi-modal imaging data, which would enable structure-by-structure, location-dependent statistical analysis. ABA is suitable for such a multi-modal approach because it can be applied to serve as the common anatomical framework (Oishi et al., 2011b, 2013; Faria et al., 2012a). The application of a multi-modal approach to the neonatal population is anticipated, as it can enhance the information available to describe the variety of anatomical features seen in neonatal brains.

### Multiple PMs and Multiple Granularity Analysis

The application of a predefined single PM has several limitations, the most important of which, perhaps, is that there are multiple criteria that can be used to define structures. For instance, when using a PM to detect pathological changes related to a specific disease, the ideal PM should define the brain structures or areas vulnerable to the disease. In diseases that affect the vasculature of the brain, a PM that is based on vascular territories might be able to quantify changes with higher sensitivity than an ontology-based brain parcellation. Application of multiple PMs with different criteria enables reasonable extraction of anatomical features. Moreover, in order to detect changes related to diseases that cause widespread pathology of the brain, a PM with lower granularity can detect such changes with higher sensitivity than a PM with higher granularity. However, a PM with higher granularity is suitable for the detection of focal changes. To extract anatomical features of various diseases, a multiple granularity approach was developed (Djamanakova et al., 2014) that can flexibly change the granularity level of the PM based on the hierarchical relationships of 254 structures (parcels) defined in the JHU-MNI-SS atlas (Oishi et al., 2009). In this multiple granularity approach, the 254 structures were dynamically combined at five different hierarchical levels, down to 11 structures, to provide a flexible view from which to extract the anatomical features of the brain. This approach is also anticipated for neonatal image analysis since various diseases can cause either widespread changes (e.g., leukodystrophies or metabolic diseases) or focal changes (e.g., focal cortical dysplasia).

### High-throughout Analysis

The ABA is an ideal method for use in the emergent big-data analysis because it can quantify and extract relevant information from medical images. The PM could compress raw images (usually more than 100 MB pixels each) into a manageable size (∼200 structural units each) in an interpretable way. High-throughput analysis is advantageous because of its potential to efficiently analyze the large amounts of imaging data using machine-learning and supervised clustering algorithms. There is also the possibility of integrating non-image related parameters. Research in the emerging field of neuroinformatics seeks to incorporate all of these data, such as those constructed in a pipeline to evaluate neuropsychiatric disorders (Miller et al., 2013) or for normal neurodevelopment in typically developing children (Jernigan et al., 2015). Particularly for the neonatal studies, in which early prediction of the later neurological, psychological, and cognitive outcomes is the desired goal, big-data analysis is essential since multiple factors, from genetics to environment, might affect the result and all of these factors need to be incorporated into the analysis.

### Clinical Applications

Atlas-based analysis has the potential to enhance the usefulness of DTI as a modality with which to predict neurological and psychiatric outcomes. However, scientific success does not necessarily guarantee clinical success. Contrary to research MRIs, clinical MRIs contain artifactural heterogeneity, which comprises variations in scan protocol and hardware performance (Back and Miller, 2014). Consequently, the image quality is usually not as stable as that of research MRIs. Moreover, there is biological heterogeneity, which comprises variations of demographics and co-morbidities, as opposed to the homogeneous research population selected through strict inclusion and exclusion criteria. In general, heterogeneity in clinical practice is one of the major causes of failure in the clinical application of scientific discovery. Extending the ABA to test its robustness in clinical scenarios, especially with age and sex-appropriate age-related growth curves for the individual brain regions, is required in the future, and is a future direction of DTI research in general.

## Conclusion

Atlas-based analysis has shown great potential for the study of neurodevelopmental patterns in normal and abnormal cohorts in the pediatric and adult populations. Currently, DTI studies incorporating ABA to study the neonatal population has demonstrated its utility in assessing neurodevelopmental progress. Further studies are needed to overcome the current limitations of ABA and incorporate it into high-throughput analyses, with the ultimate goal of application to the clinical setting.

## Conflict of Interest Statement

The authors declare that the research was conducted in the absence of any commercial or financial relationships that could be construed as a potential conflict of interest.

## Footnotes

1 http://www.bic.mni.mcgill.ca/ServicesAtlases/HomePage

2 http://www.loni.usc.edu/atlases/

3 http://fsl.fmrib.ox.ac.uk/fsl/fslwiki/Atlases

4 http://www.loni.usc.edu/atlases/

5 http://www.med.unc.edu/bric/ideagroup/free-softwares

6 http://martinos.org/lab/lcn/resources

7 http://www.fz-juelich.de/inm/inm-1/EN/Home/

8 http://cmrm.med.jhmi.edu

9 https://www.mristudio.org

10 http://www.loni.usc.edu/atlases/

11 http://cmrm.med.jhmi.edu
